# Molecular Characterization of Seminoma Utilizing the AACR Project GENIE: A Retrospective Observational Study

**DOI:** 10.3390/cancers17203363

**Published:** 2025-10-18

**Authors:** Suchit R. Geereddy, Amber Chang, Alma Gallegos, Jonathan Lin, Akaash Surendra, Suraj Puvvadi, Beau Hsia, Abubakar Tauseef, Joseph Thirumalareddy, Akshat Sood

**Affiliations:** 1 College of Biological Sciences, University of California–Davis, Davis, CA 95616, USA; allgallegos@ucdavis.edu; 2 College of Medicine, University of South Florida Health Morsani, Tampa, FL 33602, USA; achang41@usf.edu; 3 College of Arts and Sciences, Santa Clara University, Santa Clara, CA 95053, USA; jlin13@scu.edu; 4 Department of Medicine, Arizona State University, Tempe, AZ 85281, USA; asurend5@asu.edu (A.S.); spuvvad1@asu.edu (S.P.); 5 School of Medicine, Creighton University, Phoenix Campus, Phoenix, AZ 85012, USA; 6 School of Medicine, Creighton University, Ohama Campus, Ohama, NE 68124, USA; abubakartauseef@creighton.edu (A.T.); josephthirumalareddy@creighton.edu (J.T.); akshatsood@creighton.edu (A.S.)

**Keywords:** seminoma, AACR project GENIE, somatic mutations, KIT/RAS/MAPK, PI3K/AKT/mTOR, biomarker discovery, targeted therapy, cancer genomics

## Abstract

Background: Seminoma is a malignant germ cell tumor that most commonly involves the testicles but may involve the mediastinum, the retroperitoneum, and other extra-gonadal sites as well. This study aims to investigate the somatic genomic landscape of seminoma. Methods: Data for a retrospective observational analysis of seminoma was acquired from the American Association for Cancer Research (AACR) Project Genomics Evidence Neoplasia Information Exchange (GENIE) with clinical and genomic data from 2017 and beyond. Using the R and R Studio software (R 4.5.0), analyses for common somatic mutations and copy number alterations were run with a statistical significance of *p* < 0.05. Results: The most mutated genes included *KIT* (22.6%), *KRAS* (17.1%), and *MTOR* (5.1%), with significant copy number alterations in *CDKN1B* (17.2%), *KRAS* (14.7%), *CCND2* (10.3%), and *H3F3C* (9.8%). These suggest involvement within the KIT/RAS/MAPK and PI3K/AKT/mTOR (PAM) pathways for seminoma development. A novel finding within comparative evaluation of *PMS1* and *AMER1* mutations were found in Black individuals. Additionally, our findings were consistent with a lower testicular cancer rate among individuals with African ancestry than European ancestry. *BRD4* mutations were found only in metastatic samples while *KMT2C*, *STAG2*, *ALK*, *AXL*, and *EGFR* were only found in primary samples, suggesting a possible association. Conclusions: This study provided a comprehensive molecular and genetic profiling of seminoma including key genetic alterations, affected pathways, and potential therapeutic strategies. Moreover, overlap between pathways and gene mutations provides the potential for alternative treatment options for seminoma via multiple pathways.

## Simple Summary

Around 50–60% of testicular cancer cases consist of seminoma: a malignant germ cell tumor primarily diagnosed in middle-aged adults. This study explores the genomic landscape of seminoma by utilizing a de-identified publicly available dataset through the American Association for Cancer Research (AACR) Project Genomics Evidence Neoplasia Information Exchange (GENIE). The goal of this study was to further the understanding behind the biological implications of seminoma through analysis of somatic alterations, copy number alterations, therapeutic strategies, and signal cascade pathways. The findings in this study propose alternative therapeutic strategies and additional biomarkers through the investigation of various pathways.

## 1. Introduction

Seminoma is a malignant germ cell tumor that most commonly involves the testicles and less frequently the mediastinum, the retroperitoneum, and other extra-gonadal sites [1]. Histopathologically, pure seminoma is classified only when no nonseminomatous factors are present [1]. Key histological features of seminoma include a diffuse arrangement of pale cells characterized by pale to clear cytoplasm, crisp cytoplasmic membranes, and large, central nucleoli divided by fibrovascular septa containing lymphocytes [1].

Clinically, a nodule or painless swelling of the testis is often present, with patients noting a dull ache or heaviness in either the lower abdominal region, perineal area, or scrotum [1]. Prognosis for testicular seminoma varies by clinical stage, with 5-year relative survival rates of approximately 94.1% for stage I, 87.5% for stage II, and 66.7% for stage III [2].

Seminomas comprise 50–60% of testicular cancer cases, with a median age of diagnosis of 35–39 years [3]. Northern European populations exhibited the highest rates of testicular cancer incidence (8.0–9.0 per 100,000) in general, while Asian and African populations exhibited the lowest rates (<1 per 100,000) [4]. Established risk factors include cryptorchidism, a previous diagnosis of testicular cancer, a genetic predisposition, and in utero estrogen exposure [3].

The initial workup for patients with a suspected testicular mass requires measurement of serum tumor marker levels, including alpha-fetoprotein (AFP), β-human chorionic gonadotropin (β-HCG), and lactate dehydrogenase (LDH) [1]. Although histology remains the truest method for confirming seminoma diagnosis, current histological and serum-level markers are unable to differentiate between subtypes of seminoma, specifically identifying tumors resistant to radiation and chemotherapy [1,5].

A scrotal ultrasonography can rule out other conditions, and after a confirmed seminoma diagnosis, scans such as chest X-ray or CT, abdominal and pelvic CT, brain MRI, and bone scan may be performed to check for cancer spread [1].

Treatment plans also depend on the tumor stage [1]. For seminoma at any stage, radical orchiectomy is the initial procedure that provides both diagnosis and treatment [1]. Subsequent treatment with chemotherapy or radiation is based on the disease’s clinical staging after the surgery [1]. Depending on lymph node involvement, radiation and/or chemotherapy may be recommended for stage II diseases, and chemotherapy is preferred for stage III, but in select cases, radiation may be added [1,6].

While the precise molecular changes that lead to this transformation remain elusive, the most frequently observed genetic alteration is isochromosome 12p (i(12p)), which involves the duplication of the p arm of chromosome 12 [1,7]. The presence of extra 12p copies suggests higher gene dosage, which can lead to the overexpression of key genes that may contribute to seminoma development [7]. Although single-gene mutations are relatively uncommon, mutations in individual genes like *BRAF*, *KIT*, *KRAS*, *NRAS*, and *TP53* have been noted in germ cell tumors [1]. Furthermore, unified molecular markers for seminoma prognosis, particularly for evaluating immunotherapy effectiveness, require further evaluation, underscoring the need for molecular and genetic profiling to improve diagnosis, guide risk, predict immunotherapy response, and identify targets [5].

Despite the high cure rates of seminoma, particularly in early stages, there is a gap in the knowledge regarding the comprehensive genomic profile of seminoma. Current research remains insufficient in discerning which demographic features are linked to the development of primary or metastatic seminoma. Furthermore, the role of specific mutations in determining these outcomes, including whether they are mutually exclusive or co-occur, warrants further study. This study aims to characterize the somatic genomic landscape of seminoma by leveraging the GENIE dataset. By differentiating seminoma from other subtypes of testicular cancer, these findings will help refine diagnostic accuracy, advance research, and enable targeted therapies, contributing to patient care.

## 2. Materials and Methods

Institutional review board approval for this study was waived by Creighton University (Phoenix, AZ, USA), as the research exclusively utilized de-identified publicly available data, and therefore did not constitute human subjects research under federal regulations. The data was obtained from the American Association for Cancer Research (AACR) Project Genomics Evidence Neoplasia Information Exchange (GENIE) [8]. This is a publicly accessible international cancer registry designed to facilitate precision oncology research through the aggregation of clinical-grade genomic and clinical data from cancer patients across multiple institutions. Data retrieval was performed on 26 June 2025, using the cBioPortal for Cancer Genomics (version 17.0-public), an open-source web-based platform that provides visualization analysis and download access to larger-scale cancer genomics datasets. The dataset included both clinical and genomic data collected and curated since 2017.

The AACR Project GENIE database represents a collaborative effort among 19 international cancer centers. This expansive dataset reflects the real-world variability found in clinical sequencing, incorporating multiple platforms, including whole-genome sequencing (WGS), whole-exome sequencing (WES), and targeted gene panels that range in size from 50 to 555 genes. Approximately 80% of samples were sequenced using targeted panels, while about 15% underwent WES and 5% were processed with WGS. As expected, sequencing depth varied across platforms: targeted panels offered the highest coverage, typically exceeding 500×, followed by WES at around 150×, and WGS at approximately 30×. In terms of sample composition, 65% were tumor-only specimens, while 35% included matched tumor-normal pairs. These paired samples are particularly valuable, as they allow for the filtering out of germline variants, helping researchers focus more precisely on somatic mutations relevant to cancer development and treatment. This heterogeneity in sequencing methods and data types is part of what makes the GENIE dataset both complex and powerful, mirroring the diversity of real-world clinical practice.

Each institution contributing to the GENIE consortium uses its own bioinformatic pipeline for identifying and annotating mutations. However, all data are standardized using GENIE’s harmonization protocols to allow for consistent analysis across institutions. This process is supported by Genome NEXUS, which incorporates commonly used tools such as GATK (v4.6.2.0) for variant calling and ANNOVAR (2 March 2025 build) for annotation, although institutions may apply different versions or customized setups. Clinical outcome and therapeutic response data are available for certain cancer types, but a notable limitation is that treatment information is not available for seminoma. Additionally, despite harmonization efforts, differences in sequencing and annotation methods may still occur both across and within institutions. Even so, GENIE maintains a careful balance between institutional flexibility and standardized data practices, enabling large-scale collaborative research.

This study focused on patients with a confirmed pathological diagnosis of seminoma. Tumor samples were classified as primary, meaning they originated from the original tumor site, or metastatic, meaning they were taken from locations where the cancer had spread. To compare mutation patterns between these two groups, a chi-squared test was used to evaluate differences in gene-specific mutation frequencies based on the proportion of mutated samples in each category.

This analysis incorporated multiple data types, including somatic mutation profiles, tumor histology classifications, and patient demographic variables such as age, sex, and racial background. Despite variability in the design of targeted sequencing panels across institutions, the majority consistently included high-priority oncogenic drivers relevant to seminoma, such as *TP53*, *PIK3CA*, and *KMT2D*. Panels generally omitted genes lacking known clinical relevance, and as such, non-actionable mutations were underrepresented. Additionally, structural alterations, such as large insertions, deletions, or rearrangements, were excluded from this study due to either panel limitations or analytical scope. Samples with missing data were also excluded from the analysis. As a result, our findings center on frequently mutated, clinically informative genes and reflect the strengths and boundaries of panel-based genomic profiling.

To characterize genomic instability, we analyzed copy number alterations (CNAs), specifically examining homozygous deletions and high-level amplifications. Frequencies of recurrent events were computed to identify commonly altered regions across the cohort. In parallel, tumor mutational burden (TMB) was quantified by calculating the number of somatic mutations, both synonymous and nonsynonymous, per megabase (Mb) of DNA sequenced. Given the variability in panel sizes used across institutions, raw TMB values were first normalized by dividing the total number of mutations by the effective panel size. To improve cross-study and cross-platform comparability, these panel-derived TMB values were further adjusted to estimate whole-exome sequencing (WES)-equivalent TMB. This was done using linear regression models developed by the AACR Project GENIE consortium, which account for panel size and other factors influencing mutation detection sensitivity. These models help address the inherent heterogeneity in targeted sequencing panels and enable more consistent interpretation of TMB across datasets generated from different platforms. The adjusted WES-equivalent TMB values are accessible upon request through the GENIE consortium.

All statistical analyses were conducted using R and R Studio (R Foundation for Statistical Computing, Boston, MA, USA). Statistical significance was defined as a *p*-value less than 0.05. Continuous variables are reported as means with standard deviations (mean ± SD), while categorical variables are presented as counts and percentages. Associations between categorical variables were evaluated using the chi-squared test. For continuous variables, normality was first assessed; group comparisons were then made using a two-sided Student’s *t*-test for normally distributed data or the Mann–Whitney U test for non-normal distributions. To account for multiple testing, *p*-values were adjusted using the Benjamini–Hochberg false discovery rate (FDR) correction.

Somatic mutations included in the analysis were filtered to retain only nonsynonymous variants, specifically missense, nonsense, frameshift, and splice-site mutations that met a variant allele frequency (VAF) threshold of 5% or higher and a minimum sequencing coverage of 100×. Synonymous mutations and variants of uncertain significance (VUS) were excluded from consideration. Mutation calls were obtained from the GENIE consortium’s harmonized Mutation Annotation Format (MAF) files, which provide standardized and uniformly curated variant annotations, including concise gene and protein alteration nomenclature, ensuring consistency across all contributing institutions (AACR Project GENIE).

## 3. Results

### 3.1. Patient Demographics of Seminoma

Given the rarity of this cancer type in genomic datasets, both primary and metastatic tumor samples were included in the demographic assessment. A total of 217 samples were analyzed from 211 patients. The patient demographics are shown in Table 1. The vast majority of patients were male, comprising 209 cases (99.1%), while no female patients were represented in the cohort. In terms of ethnicity, 170 individuals (80.6%) were identified as non-Hispanic, 14 (6.6%) were Hispanic, and ethnicity was either unknown or not reported for 27 cases (12.8%). The racial composition was predominantly White, accounting for 170 patients (80.6%), followed by 12 (5.7%) identifying as Other, 8 (3.8%) as Asian, and 3 (1.4%) as Black. Race information was missing or unspecified in 18 cases (8.5%). Sample type varied, with 136 tumor samples (62.7%) obtained from primary tumors, and 70 (32.3%) collected from metastatic tumors. For 7 samples (3.2%), the site of origin was not recorded. Regarding the age category, 209 adults were captured in the dataset.

**Table 1:** Seminoma patient demographics.

Demographics	Category	*n* (%)
Sex	Male	209 (99.1%)
	Female	0 (0.0%)
	Not Reported	2 (0.9%)
Age Category	Adult	209 (99.1%)
	Pediatric	0 (0.0%)
	Not Reported	2 (0.9%)
Ethnicity	Non-Hispanic	170 (80.6%)
	Unknown/Not Collected	27 (12.8%)
	Hispanic	14 (6.6%)
Race	Asian	8 (3.8%)
	White	170 (80.6%)
	Black	3 (1.4%)
	Other	12 (5.7%)
	Unknown	18 (8.5%)
Sample Type	Primary	136 (62.7%)
	Metastasis	70 (30.3%)
	Not Collected	7 (3.2%)
	Unspecified	4 (1.8%)

### 3.2. Most Common Somatic Mutations and Copy Number Alterations (CNAs)

**Figure 1:**
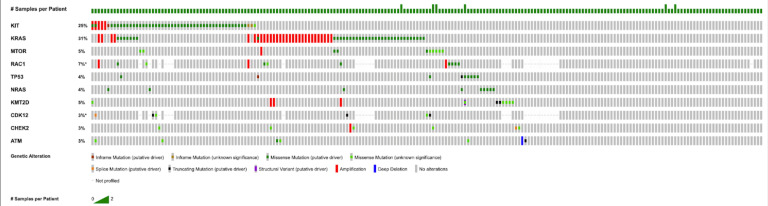
OncoPrint displays recurrent mutations in seminoma observed in at least 5 cases (*n* ≥ 5). This is with sequencing coverage of at least 100× (≥100×) and VAF of at least 5% (VAF ≥ 5%). Asterisks indicate sampling with incomplete profiling.

Figure 1 highlights the most frequently altered genes within this 217-person cohort. The gene *KIT* showed the highest mutation rate (*n* = 49, 22.6%), followed by *KRAS* (*n* = 37, 17.1%). Other commonly mutated genes included *MTOR* (*n* = 11, 5.1%), *RAC1* (*n* = 9, 4.1%), *TP53* (*n* = 9, 4.1%), *NRAS* (*n* = 9, 4.1%), and *KMT2D* (*n* = 9, 4.1%), as well as *EP300* (*n* = 7, 3.2%). Less frequent but still notable were *CDK12* (*n* = 6, 2.8%), *CHEK2* (*n* = 6, 2.8%), and *ATM* (*n* = 6, 2.8%). Overall, mutations in genes associated with cell signaling and regulation were recurrent, with *KIT* and *KRAS* standing out as the most significantly altered in this dataset.

Beyond point mutations, analysis of CNAs in 204 profiled samples revealed several recurrent gene amplifications, especially among proto-oncogenes. The most prominent was *CDKN1B* (*n* = 35, 17.2%), followed by *KRAS* (*n* = 30, 14.7%). Other genes frequently showing copy number gains included *CCND2* (*n* = 21, 10.3%), *H3F3C* (*n* = 20, 9.8%), *ETV6* (*n* = 19, 9.3%), *RAD52* (*n* = 19, 9.3%), and *PIK3C2G* (*n* = 17, 8.3%). These amplifications often affect genes involved in cell cycle regulation, growth signaling, and DNA repair, suggesting a role in oncogenic progression.

### 3.3. Genetic Differences by Race and Sex

In this cohort, several gene mutations were significantly enriched in Black patients. For example, *PMS1* and *AMER1* mutations were exclusively found in Black individuals (*n* = 1 each), with *PMS1* showing significance (*p* = 0.0000) and *AMER1* having an extremely low *p*-value (*p* < 0.001; *q* = 1.64 × 10^−9^) (Date collected: 28 June 2025). Similarly, *RB1* mutations occurred in one Black and one White individual but were most enriched in Black patients (*p* < 0.001; *q* = 4.96 × 10^−5^). Mutations in *PIK3CD*, *EPHA5*, *TCF3*, *FH*, *BCL2L1*, and *KEL* demonstrated significant enrichment among Black patients (*p* < 0.05). In comparison, *POLE* and *MAP3K4* mutations were more frequent in the “Other” racial group (*n* = 2 and *n* = 1, respectively; *p* < 0.001, *q* = 3.08 × 10^−4^). Alterations in *BORCS8-MEF2B*, *MKRN1*, *and CIC* were detected exclusively in Asian patients, each showing statistically significant enrichment (*p* < 0.001; *q* range 7.31 × 10^−4^ to 2.52 × 10^−3^). Lastly, a *NUP214* mutation was identified in a single White individual (*n* = 1; *q* = 7.31 × 10^−4^). The differences in recurrent mutations among Black, Asian, and White patients are displayed in Table 2.

**Table 2:** Race and associated mutations.

Gene (Chi-Squared)	Black, *n* (%)	Asian *n* (%)	White, *n* (%)	*p* Value
AMER1	1 (33.33)	0 (0.0)	0 (0.0)	*p* < 0.001
PMS1	1 (50.00)	0 (0.0)	0 (0.0)	*p* < 0.001
PIK3CD	1 (50.00)	0 (0.0)	0 (0.0)	*p* < 0.001
BORCS8-MEF2B	0 (0.0)	1 (12.50%)	0 (0.0)	*p* < 0.001
MKRN1	0 (0.0)	1 (12.50%)	0 (0.0)	*p* < 0.001
NUP214	0 (0.0)	0 (0.0)	1 (100.00)	*p* < 0.001
CIC	0 (0.0)	1 (12.50%)	0 (0.0)	*p* < 0.001

### 3.4. Co-Occurrence and Mutual Exclusivity of Mutations

In this dataset, no statistically significant patterns of gene co-occurrence or mutual exclusivity were identified. In this dataset, the association between MTOR and CDK12 (*n* = 2/13; *p* = 0.027) showed statistically significant co-occurrence, while the association between TP53 and CDK12 (*n* = 1/10; *p* = 0.162) was close to significance. Similarly, potential mutual exclusivity was suggested between *KRAS* and *NRAS (p* = 0.277) and *KIT* and *KMT2D* (*p* = 0.301), but these associations did not reach statistical significance. Overall, the data does not reveal strong or consistent gene-pairing trends in this cohort.

### 3.5. Primary vs. Metastatic Mutations

A total of 217 tumor samples were analyzed, comprising 70 metastatic and 136 primary tumors. Several gene-level differences were observed between these groups. *BRD4* mutations were present in 6 metastatic cases (8.7%) and absent in primary tumors (*p* = 0.00125), suggesting a possible association with metastasis. Similarly, mutations in *KMT2C*, *STAG2*, *ALK*, *AXL*, and *EGFR* were observed only in metastatic samples, though the small sample sizes limit definitive conclusions. Conversely, *KIT* mutations were more frequent in primary tumors (30.9%) compared with metastatic cases (11.4%), with statistical significance (*p* = 0.00191). Overall, while most alterations overlapped between primary and metastatic tumors, the enrichment of *BRD4* in metastatic disease and *KIT* in primary tumors suggest potentially meaningful differences in progression that should be confirmed in independent datasets.

## 4. Discussion

### 4.1. Subgroups and Mutational Landscape

This study aimed to characterize the genomic landscape of seminoma using the data from the AACR Genie dataset. Thorough review of primary and metastatic tumor samples revealed a multitude of gene alteration patterns across different patient subpopulations. Considering the global incidence patterns, the racial makeup of this cohort was predominantly white (*n* = 170), aligning with the established incidence of seminoma amongst non-Hispanic whites within most studies [9,10,11]. Comparative evaluation within the cohort found mutually exclusive *PMS1* and *AMER1* mutations (*n* = 1 each) in Black individuals. This represents a novel finding as no prior studies have focused on the association between aforementioned mutations and Black seminoma patients. However, beyond gene alterations, African-ancestry men are less likely to develop testicular cancer compared to European-ancestry men [12], supporting our cohort resulting in a low frequency of Black patients in our study (*n* = 3).

### 4.2. Commonly Mutated Genes and Altered Pathways

In agreement with prior studies, the seminoma cohort has shown genetic heterogeneity with many varying genetic mutations that contribute to a multitude of pathways such as cell cycle regulation, growth signaling, and DNA repair, suggesting a role in oncogenic progression. This study identified many gene mutations, most frequently affecting the PI3K/AKT/mTOR pathway through *MTOR* (5.1%), *PIK3C2G* (8.3%), and *KRAS* (14.7%) [13,14,15,16,17,18,19,20,21,22]. Additional gene mutations, consistent with prior literature, partaking in the cell cycle and DNA repair include *CDKN1B* (17.2%), *CCND2* (10.3%), *TP53* (4.1%), *ATM* (2.8%), *CHEK2* (2.8%), and *RAD52* (9.3%) [23,24,25,26]. Furthermore, mutations part of the KIT/RAS/MAPK pathway were present as well via *KIT* (22.6%), *KRAS* (17.1%), *NRAS* (4.1%), and *RAC1* (4.1%) [27,28,29,30,31,32]. Other gene alterations include *RAC1* (4.1%), *KMT2D* (4.1%), *EP300* (3.2%), *CDK12* (2.8%), *H3F3C* (9.8%), and *ETV6* (9.3%). These findings align with former studies analyzing *KIT*, *KRAS*, and *CDKN1B* alterations in seminoma, along with their roles in the MAPK/ERK pathway, PI3K/AKT pathway, and cell cycle regulation [13,27,28,29,33,34,35]. This further supports the evidence that they serve a crucial role in putative oncogene drivers.

Standard treatments for seminoma involve the use of radical inguinal orchiectomy at any stage, although 15% of patients relapse after >3 years [6,36,37]. Adjuvant radiotherapy has historically proven to reduce the relapse in clinical stage (CS) I disease, with recurrence rates dropping to 1.4–6.9% [6]. In more advanced stages (CS IIA/IIB), platinum-based combination chemotherapies, such as bleomycin, etoposide, and cisplatin (BEP), or radiotherapy can be utilized [36,37]. In a study conducted by Domont et al., the 67-patient cohort of stage II seminoma had an overall survival rate of 97% after treatment [38]. While platinum-based chemotherapy remains highly effective, it does pose its own array of barriers, including treatment-induced toxicities, such as secondary malignancies and cardiovascular disease, and platinum-resistant cases associated with *KIT* and *RAS* mutations [6,24,36,37]. With patients who experienced refractory or relapsed disease, it is followed by second- and third-line (salvage) chemotherapies, progressing to high-dose chemotherapy with autologous stem-cell transplant (ASCT) [39,40]. Among the options, CLDN6-targeted antibody-drug conjugates (ADCs) and CAR T-cell therapies are emerging, although they are early in clinical trials.

Our analysis did not reveal a single distinct mutation or hotspot specific to seminoma, consistent with previous studies that highlight a polygenic model with *KIT* and *KRAS* as frequent drivers [34]. These heterogeneous mutation patterns are supported by prior studies that highlight a polygenic model rather than a monogenic model in regard to signaling pathways in TGCTs [16,33,36]. This diversity in genetic patterns hardens the search for therapeutic treatments but allows for exploration in a wide spectrum of potential receptiveness.

### 4.3. KIT/RAS/MAPK Pathway

The KIT protein is a receptor tyrosine kinase (RTK) that activates cell proliferation and differentiation through the activation of both RAS/MAPK and PI3K/AKT pathways [23,27,29,30]. We found *KIT* gene mutations at the highest rate of the RAS pathways (22.6%), followed by *KRAS* (17.1%) and *NRAS* (4.1%). Our percentage of *KIT* falls within previously reported mutation rates with prior studies regarding TGCT that range between 8.1 and 25.9% [29,30,31]. *KIT* mutations are identified as early driver mutations during embryonic development and are associated with progression of diseases [27]. Combination therapies, such as imatinib mesylate and third-line chemotherapy regimen, can potentially be used to target through downstream effects on other pathways, like the PI3K/AKT pathway, via the shared *KIT* intermediate [41]. A case study conducted by Pectasides et al. using the aforementioned therapy treatment achieved a complete response from a chemo resistant stage IV seminoma patient suggesting a multi-pathway impact through *KIT* [41]. While there is limited testing on seminoma, it provides a path for seeking alternative treatment options via multiple pathways.

### 4.4. PI3K/AKT/mTOR (PAM) Pathway

The PAM pathway is one of the most commonly dysregulated pathways in oncology, occurring in around 50% of tumors [14]. By compiling the results from our frequently altered genes, the highest mutation rates in genes associated with the PAM pathway were *MTOR* (5.1%), *RAC1* (4.1%), and *TP53* (4.1%), with *PIK3C2G* accounting for 8.3% of CNAs, consistent with previous studies conducting extensive research into *MTOR* and *RAC1* in testicular tumors and solid tumors, respectively [16,32,42]. While seminoma-specific data is scarce, studies [14,15,21,22] have shown the PAM pathway as a progression driver for malignant proliferation through various mechanisms of activation, despite activation by *KIT* in part. In a study by Yaba et al., mTOR expression in seminoma makes it a potential target for new treatments and emphasizes the dependence that seminoma, and other cancers, have on the PAM pathway [17]. As for therapeutic methods, cascade signaling inhibitors used alongside conventional methods, such as rapamycin and AZD8055 in conjunction with cisplatin treatment, have shown strong pathway inhibition in early clinical stages for treating seminoma [17,18,19,20,22]. A study by Onel et al. utilized rapamycin treatment to investigate its effect on mTOR signaling pathway proteins within the TCam-2 cell line. They found extensive promise in targeting the cell line using rapamycin since, at 1000 nM, it significantly promoted G1 arrest compared to the control group (no rapamycin) [18] Another literature by Rosas-Plaza et al. found that combining AZD8055 and cisplatin significantly increases apoptosis/cell death compared to cisplatin alone in testicular cancer cell lines’ 833KE, Tera, and NCCIT [19].

### 4.5. Co-Occurrence Patterns and Functional Implication

We observed trends suggesting potential mutual exclusivity between *KRAS* and *NRAS* (*p* = 0.277) as well as *KIT* and *KMT2D* (*p* = 0.301), although these findings did not reach statistical significance. They align with prior studies in TGCTs, where *KIT*, *NRAS*, and *KRAS* are found to be mutually exclusively mutated [23,34,43]. There are no well-documented studies that display the association between *KIT* and *KMT2D* as a chromatin modifier gene and oncogene, respectively.

As for co-occurrence, there are preceding studies regarding *CDK-12* and *TP53* or *CDK-12* and *MTOR*, specifically toward seminoma or TGCT. In our data, both *MTOR* and *CDK-12* (*p* = 0.027) as well as *TP53* and *CDK-12* (*p* = 0.162) displayed tendencies towards co-occurrence. In regard to *CDK-12* and *TP53*, prior findings have shown that combined inactivating mutations resulted in accelerated prostate tumorigenesis compared to each event alone [25]. *CDK-12* and *MTOR* have shown cooperation in affecting translation of mRNA that are responsible for DNA repair factors and translation factors [26]. These consistencies underscore the extensive network of seminoma that is made up of a wide array of gene mutations and influenced by each other or environmental factors, justifying extensive research into global incidences.

### 4.6. Primary vs. Metastatic Samples

Notable differences were observed through the assessment of primary and metastatic samples of seminoma, revealing *BRD4* mutations were overrepresented in metastatic cases (*p* = 0.00125), while *KIT* mutations were more common in primary samples (*p* = 0.00191). These findings are consistent with the prior studies conducted around TGCTs and seminoma [27,44] which reported *BRD4* and *KIT* found in metastatic and primary samples, respectively. The enrichment of *BRD4* in metastases suggests that it may be a factor in increasing cell proliferation and enhanced cell survival, as it has shown its capabilities along prostate cancer lines [45].

### 4.7. Limitations

There are several limitations that come with this study. First, there is no treatment information in this database for us to analyze the response to treatments with the genetic mutation profile and histologic classification. Additionally, this obstructs primary and metastatic tumor comparisons through therapy-related genomic alterations. Second, the AACR Project GENIE database lacks both transcriptomic data and miRNA data. The transcriptomic data pertains to seminoma as it can define the origin, emphasize active pluripotency, and explain the developmental context of seminoma [46]. Absence of this data in our set prevents the correlation between mutations and downstream expression. In parallel, miRNA data can provide insight into the distinct miRNA signature that seminoma provides [47]. Third, linking genetic mutations and patient outcomes or seminoma’s unique disease aspects is constricted through the sample size, preventing statistical reliability. In order to ensure that specific mutations can predict a patient’s prognosis consistently, more research must be conducted using a larger sample size of patients with reliable clinical and genomic data. Fourth, the study design hinders the separation between notable driver mutations and passenger mutations that occur through tumor development, as it lacked longitudinally collected samples (i.e., matching sample/metastatic samples).

Fifth, the repository utilized multiple sources to compile their data via an array of sequencing platforms that may introduce discrepancies leading to bias in mutation rates. Sixth, this study did not consider the sway that DNA methylation or epigenetic control has within seminoma. Moreover, the consequences affect the treatment response and behavior of the tumor as they were not taken into account. Otherwise, this would provide valuable genetic information for a better understanding. Seventh, the lack of clinical outcomes within our dataset obstructed the linkage between mutations and survival outcomes (both disease-free and overall). Eighth, acknowledgement of the data that may be skewed as some samples are not independent in the GENIE repository (i.e., samples from the same patient). However, this plays a minor role in the primary outcomes of the study.

Ninth, all the seminoma histological subtypes (classical, anaplastic, spermatocytic) are grouped into one overall group. This hinders analysis of clinical outcomes and their specific subtype mutational profiles. Further research must be conducted in the future to strengthen the association between genomic mutations and clinical data to longitudinally confirm the impact of all of the observed mutations on patients with seminoma. Along with this, an additional constraint affects the linkage between tumors’ protein expression and genetic mutations due to the inability to explore them through immunohistochemistry. The current analysis produces important information regarding the profile of genomic alterations typically found in seminoma, emphasizing the significance of known pathways such as MAPK, PI3K, and cell cycle, and finding new targets for therapeutic intervention while recognizing the limitations. Future research should further validate these findings, expand on clinical relevance, and assess value in diverse populations. These efforts are crucial in turning genomic interpretations into diagnostic, prognostic, and therapeutic strategies for patients with seminoma.

Some strengths can be highlighted by leveraging the AACR Project GENIE database; this study benefits from one of the largest available real-world genomic datasets. This provides broader representation across institutions, sequencing platforms, and patient populations compared to single-institution studies. Seminoma is relatively rare, making it challenging to gather sufficiently powered datasets. This study adds meaningful genomic data to a cancer type where molecular characterization is less well-documented, helping fill an important gap in TGCT research.

## 5. Conclusions

In summary, analysis of AACR Project GENIE data sheds light on a distinct seminoma genomic profile characterized by recurrent mutations in *KIT*, *KRAS*, and *MTOR*, as well as focal copy number alterations in *CDKN1B*, *KRAS*, *CCND2*, and *H3F3C*. The pattern of alterations observed suggests involvement of the KIT/RAS/MAPK and PI3K/AKT/mTOR (PAM) signaling pathways in seminoma development. Notably, we observed clear genomic differences across race and sex, supporting the use of more personalized therapeutic approaches for patients. These results advance our understanding of seminoma molecular drivers and suggest specific pathways that warrant further preclinical and clinical evaluations as targeted therapy options.

## Data Availability

The data presented in this study are available from the AACR GENIE Database at https://genie.cbioportal.org/ (accessed on 26 June 2025).

## Abbreviations

The following abbreviations are used in this manuscript:

AACR	American Association for Cancer Research
ADC	Antibody-Drug Conjugate
AFP	Alpha-Fetoprotein
*ALK*	Anaplastic Lymphoma Kinase
*AMER1*	APC Membrane Recruitment Protein 1
ASCT	Autologous Stem-Cell Transplant
*ATM*	Ataxia Telangiectasia Mutated
BEP	Bleomycin, Etoposide, and Cisplatin
*CDK12*	Cyclin-Dependent Kinase 12
*CDKN1B*	Cyclin-Dependent Kinase Inhibitor 1B
CEB	Carboplatin, Etoposide, and Bleomycin
*CHEK2*	Checkpoint kinase 2
CNAs	Copy Number Alterations
CS	Clinical Stage
DNA	Deoxyribonucleic Acid
*EGFR*	Epidermal Growth Factor Receptor
*EP300*	E1A Binding Protein P300
ERK	Extracellular Signal-Regulated Kinase
*ETV6*	ETS Translocation Variant 6
FDR	False Discovery Rate
*FH*	Fumarate Hydratase
GATK	Genomic Analysis Toolkit
GENIE	Project Genomics Evidence Neoplasia Information Exchange
i(12p)	Isochromosome 12p
*KMT2C*	Lysine (K)-Specific Methyltransferase 2C
*KMT2D*	Lysine Methyltransferase 2D
LDH	Lactate Dehydrogenase
MAF	Mutation Annotation Format
MAPK	Mitogen-Activated Protein Kinase
Mb	Megabase
miRNA	MicroRNA
*mTOR*	Mammalian Target of Rapamycin
*NRAS*	Neuroblastoma RAS Viral Oncogene Homolog
PAM	PI3K/AKT/mTOR pathway
PI3K	Phosphoinositide 3-Kinase
PI3K/AKT/mTOR	Phosphoinositide 3-kinase/Protein Kinase B/Mammalian Target of Rapamycin
*PIK3C2G*	Phosphatidylinositol-4-Phosphate 3-Kinase, Catalytic Subunit Type 2 Gamma
*PIK3CA*	Phosphatidylinositol-4,5-Biphosphate 3-Kinase, Catalytic Subunit Alpha
*PIK3CD*	Phosphatidylinositol-4,5-Biphosphate 3-Kinase, Catalytic Subunit Delta
RTK	Receptor Tyrosine Kinase
SD	Standard Deviation
*STAG2*	Stromal Antigen 2
*TCF3*	Transcription Factor 3
TGCT	Testicular Germ Cell Tumors
TMB	Tumor Mutational Burden
*TP53*	Tumor Protein p53
VAF	Variant Allele Frequency
VUS	Variants of Uncertain Significance
WES	Whole-Exome Sequencing
WGS	Whole-Genome Sequencing
β-HCG	β-Human Chorionic Gonadotropin
